# Associations of the Initial COVID-19 Lockdown on Self-Reported Happiness and Worry about Developing Loneliness: A Cross-Sectional Analysis of Rural, Regional, and Urban Australian Communities

**DOI:** 10.3390/ijerph18189501

**Published:** 2021-09-09

**Authors:** Vivian Isaac, Teresa Cheng, Louise Townsin, Hassan Assareh, Amy Li, Craig S. McLachlan

**Affiliations:** 1 Flinders Rural Health South Australia, Flinders University, Renmark, SA 5341, Australia; vivian.isaac@flinders.edu.au; 2 Centre for Healthy Futures, Torrens University Australia, Pyrmont, NSW 2009, Australia; teresa.cheng@Torrens.edu.au (T.C.); craig.mclachlan@Torrens.edu.au (C.S.M.); 3 Research Office, Torrens University Australia, Adelaide, SA 5000, Australia; louise.townsin@Torrens.edu.au; 4 School of Health, Federation University Australia, Berwick, VIC 3806, Australia; 5 Evidence Generation and Dissemination, Agency for Clinical Innovation, St Leonards, NSW 2065, Australia; hassan.assareh@health.nsw.gov.au; 6 Department of Pharmacy & Biomedical Sciences, La Trobe University, Bendigo, VIC 3550, Australia

**Keywords:** COVID-19 lockdown, general population, happiness, anxiousness, loneliness

## Abstract

Australia adopted hard lockdown measures to eliminate community transmission of COVID-19. Lockdown imposes periods of social isolation that contributes to increased levels of stress, anxiety, depression, loneliness, and worry. We examined whether lockdowns have similar psychosocial associations across rural and urban areas and whether associations existed between happiness and worry of loneliness in the initial wave of the COVID-19 pandemic in Australia. Data were collected using the “COVID-19 Living Survey” between 13 and 20 May 2020 by BehaviourWorks Australia at the Monash Sustainable Development Institute. The mean self-reported feeling of happiness and anxiousness (N = 1593), on a 10-point Likert scale with 0 being least happy or highly anxious, was 6.5 (SD = 2.4) and 3.9 (2.9), respectively. Factors associated with happiness were older age and having a postgraduate education. Participants worried about becoming lonely also exhibited reduced happiness (estimate = −1.58, 95%CI = −1.84–−1.32) and higher anxiousness (2.22, 1.93–2.51) scores, and these conditions remained associated after adjusting for demographics. Interestingly, worry about loneliness was greater in rural areas than in urban communities. The negative impact of the COVID-19 lockdown on rural youth and those less-educated was evident. Participants in rural Australia who were worried about becoming lonely were reportedly less happy than participants in major cities. This dataset provides a better understanding of factors that influence psychological well-being and quality of life in the Australian population and helps to determine whether happiness may be an associative factor that could mitigate self-feelings of anxiety and worry about loneliness.

## 1. Introduction

The COVID-19 pandemic has had a significant social, economic, and psychological impact on the daily lives of people across the world. In March 2020, the Australian government introduced quarantine and contact-tracing measures to eliminate community transmission; then, with the growth of cases beyond contract tracing, a nation-wide lockdown ensued. In late June 2021, a second wave of coronavirus, the Delta strain, became predominant in urban, regional, and rural areas of New South Wales, requiring a “hard” lockdown that is currently ongoing. It is indeed interesting to explore the historical impact of lockdowns across regions of Australia to better understand the health and wellbeing associations in future lockdowns.

A hard lockdown imposes Australian state and international border closure to all non-residents, and all non-essential services, including schools, public gatherings, and events are cancelled or significantly restricted. The ultimate outcome of a hard lockdown is the implementation of strict stay-at-home requirements, with permission given to use listed essential services [1,2]. Lockdown imposes periods of social isolation that may contribute to increased levels of anxiety and loneliness [3,4,5]. Loneliness, while not caused by being alone, is defined as the perception of a current or future lack of social relations and/or absence of affection within social relationships [6,7,8]. Self-perceived loneliness is a significant indicator of social well-being and is also believed to be higher in states of anxiety and depression [6,9]. Whether happiness is independent or associated with these factors has not been previously reported in the context of regionality, age, and gender in Australia.

Happiness and anxiety are affective responses that serve as common constructs for the assessment of psychological well-being and life satisfaction [10]. Recently, the OECD’s better life index equated happiness with overall life satisfaction that is positively influenced by health, well-being, and quality of life (https://www.oecdbetterlifeindex.org/, accessed on 6 May 2021). In contrast, anxiety can be associated with worry and the development of depression. Indeed, anxiety and worry can negatively impact life satisfaction and quality of life [11]. The perceived levels of happiness and anxiety are important predictors of mental well-being and overall quality of life, yet they have received little attention with respect to their consistency across rural and urban environments and across the adult lifespan during a COVID-19 hard lockdown.

In this study, we conducted a cross-sectional analysis of the Australian population to determine factors that are associated with self-reports of happiness and worry about becoming lonely, towards the end of the first COVID-19 lockdown between 13 and 20 May 2020. It remains unknown whether worrying about becoming lonely can reduce happiness and increase anxiety. While happiness may not mitigate the worry of becoming lonely, addressing negative cognitions may lead to improved happiness. Our aims were to determine, firstly, whether stay-at-home orders have the same psychosocial associations across urban, regional, and rural areas, and, secondly, to determine whether associations exist between worry about becoming lonely and happiness during the initial COVID-19 pandemic in Australia.

## 2. Materials and Methods

### 2.1. Data Source

Data were collected via an online “COVID-19 Living Survey” as a part of the Survey of COVID-19 Responses to Understand Behaviour (SCRUB) project developed and conducted by BehaviourWorks Australia at Monash Sustainable Development Institute between 13 May and 20 May 2020. Participants aged 18 years and over were invited to complete the online survey via several paths of recruitment, external panel/participant recruitment provision, and snowball sampling (via email and social media platforms). The survey aimed to collect individual experiences with COVID-19 to inform actions taken in response to the initial COVID-19 pandemic and lockdown. Therefore, self-assessment would be framed in the context of the initial COVID-19 lockdown. The survey measured psychosocial behaviours, aspects of mental health, COVID-19 attitudes and beliefs, and demographic variables. Data were anonymized at the time of collection. At the time of data analysis, records from 1593 adult individuals were included in our analysis (See Table 1 for sample characteristics). Survey protocol, questionnaires, collection protocols, and data availability are described in detail in [12,13,14]. Ethics was approved by the Monash University Human Research Committee, ID: 23584.

### 2.2. Outcomes and Covariates

We focused on self-reported single-item measures of happiness and anxiety, which were collected based on a 10-point Likert scale from agree “not at all” to agree “completely”. We examined the associations of self-reported “worrying about becoming lonely” and rurality of participants’ area of residence on the measured outcomes while controlling for potential confounding demographics, including age, sex, and level of education. Worriedness about becoming lonely (referred to as “loneliness” in Tables 2 and 3) was collected based on a 7-point Likert scale from “Don’t worry at all” (0) to “Worry a lot” (7). Rurality was categorized based on the postcode provided as identified in the Modified Monash Model (MM), which defines whether a location is a city, rural, remote, or very remote on a scale of 1–7 according to geographical remoteness and population size (for further detail, see [15]). Outcome measures were treated as continuous; age was categorized in 10-year intervals from 18–29 years old up to age 79, and then a final category of 80 years and over; education level was categorized into high school, undergraduate (and other equivalent degrees), and postgraduate (PhD and Master degrees); rurality was recategorized into MM1 (urban/major city), MM2 (regional), MM3-4 (rural), and MM5-7 (remote) [15]; and worrying about becoming lonely was dichotomized as worry (scores between 1–4) and not worry (scores between 5–7). Between 41 to 79 records had missing covariates and subsequently were excluded across the models.

### 2.3. Data Analysis

We used descriptive statistics to summarize the demographic data and employed general linear regression modelling to estimate adjusted associations of factors of interest on the outcomes described above. Three set of models were constructed to investigate factors associated with happiness (Table 2) and anxiety (Table 3). Model 1 included demographic variables “only”. Model 2 investigated the associations of “worried about becoming lonely” and its impact on happiness/anxiety during the COVID-19 pandemic after controlling for demographic factors. In Model 3, the interaction between worried about becoming lonely and rurality was included to examine associations between them. Multicollinearity in the models with all possible covariates (Model 3) was examined and found to be negligible.

Differences were considered significant when *p* < 0.05. Statistical analyses were performed in R package, version 4.0.2 (R Core Team (2018). R: A Language and Environment for Statistical Computing (Version 4.0.2). Vienna, Austria: R Foundation Statistical Computing. Retrieved from www.R-project.org, accessed on 4 May 2021).

## 3. Results

The total sample population used in the analysis was 1593, of whom 51.8% were female. Table 1 illustrates the demographic data of our sample population. The median age of the sample was 45 with an interquartile range of 31–62. One-fourth (26.8%) of the cohort had completed their high school education, and the remaining 70.9% completed either an undergraduate or postgraduate degree, indicative of a high degree of literacy in our sample population. One-fifth (20%) of the participants represented regional, rural, or remote Australia.

Across the respondents, the mean feeling of happiness on a 10-point Likert scale was 6.5 (SD = 2.4). Table 2 identifies factors associated with the reported feeling of “happiness” during the COVID-19 pandemic. Model 1 demonstrated the association of demographic variables on feeling happy during COVID-19. Older participants in the 60–69 (estimate = 1.04, 95% CI: 0.63–1.46), 70–79 (1.66, 1.22–2.10), and over-80 (1.73, 1.01–2.45) age groups reported feeling happier compared to their younger counterparts, indicating that age may be a predictor of happiness during periods of pandemic. Higher education was also indicative of the feeling of happiness during COVID-19. Participants who received a postgraduate education reported a higher incidence of feeling happy compared to those who only received a high school education (0.59, 0.19–0.99). Geographical location did not significantly impact participant reports of happiness in Model 1.

In Table 2, Model 2 investigated the associations between “worried about becoming lonely” and its impact on happiness during the COVID-19 pandemic after controlling for demographic factors. Worry about becoming lonely during the pandemic was significantly and inversely associated with the feeling of happiness in our cohort. A high score on being worried about loneliness remained associated with reduced perception of happiness (−1.58, −1.84–−1.32) after adjusting for demographic variables.

The interaction between loneliness and geographical location was investigated in Model 3 of Table 2. Worrying about loneliness had greater impact on happiness in participants from regional and remote regions compared to major cities. That is, participants in regional and remote Australia who were worried about becoming lonely were reportedly less happy than participants in the major cities who also worried of becoming lonely during the pandemic.

Across the respondents, the mean feeling of anxiousness on a 10-point Likert scale was 3.9 (SD = 2.9). Table 3 identifies factors associated with feeling anxious during the COVID-19 pandemic. Table 3, Model 1 illustrates an association between age and anxiety during the pandemic. Consistent with the “happiness” findings (Table 2), older participants in the 50–59 (−0.85, −1.31–−0.38), 60–69 (−1.37, −1.85–−0.89), 70–79 (−1.88, −2.39–−1.36), and over-80 (−1.08, −1.93–−0.24) age groups reported comparatively less anxiety than the younger 18–29-year-old cohort. There was no association between gender and education on feeling of anxiousness during the pandemic. Furthermore, participants who reported that they were worried about becoming lonely were strongly associated with the feeling of anxiety (2.22, 1.93–2.51) in Model 2, Table 3. The association between worry about becoming lonely and anxiety were independent of age, gender, education, and geographical location (Model 2, Table 3). There was no interaction between worrying about loneliness and geographical location (Model 3, Table 3).

## 4. Discussion

This study is the first, to our knowledge, to report on the sociodemographic factors that influenced feelings of happiness and anxiety at the end of Australia’s first COVID-19 lockdown. Our primary finding was the negative impact of the COVID-19 lockdown on social cognitions of happiness in rural youth and those less educated.

Our results demonstrate that age is negatively associated with happiness. Specifically, by the end of the first wave of lockdown, we found that the rural-based young-to-middle aged adults (individuals between the ages of 18–50) in our cohort were more likely to experience lower levels of happiness and greater levels of anxiety in comparison to our older age groups. We speculate that a probable reason for the lack of happiness in our rural cohort and those in younger age groups is that they both lack emotional resilience [16] and were experiencing stressors associated with loss of work and lockdown. According to the OECD, young adults in Australia were at greatest risk of joblessness and poverty relative to their older counterparts [17]. As a result of COVID-19 job-related disruptions, 36% of 18–29-year-olds and 33% 30–49-year-olds, compared to 26% 50–69-year-olds, reported financial difficulties, with approximately one in five young adults taking money out of savings or selling assets to pay for usual expenses, such as rent, mortgage, and utilities [18]. Young individuals in regional and rural areas are faced with even greater challenges related to lower employment opportunities and increasing cost of living in these areas compared to major cities [19]. Thus, the reduced happiness and increased anxiety levels experienced by the younger and rural cohorts may be attributed to stress factors introduced by lockdowns that are external and outside of their control.

These findings were intriguing, as the adults over 50 years old observed in our study demonstrated higher levels of happiness. We had expected older adults, often with a higher incidence of chronic conditions, to report experiencing greater levels of psychological distress, such as vulnerability and anxiety, than young-to-middle aged adults given their increased risk of morbidity and mortality associated with contracting COVID-19 [20]. The results of our study are similar to recent reports out of Canada [21] and the United States [22], where older age seemingly moderates the impact of stress, anxiety, and depression. There are several hypotheses that may contribute to the buffered psychological impact associated with older age, including emotional resilience [23,24] and a shift from active to passive emotions [25,26]. Older adults are more likely to have accumulated major life experiences (e.g., post-WW2 era, global recessions, loss of loved ones) and have therefore developed coping strategies that enhance their resilience in response to external events to an extent that younger adults have not [23,24,27]. Older adults were also found to have developed a passive emotional response, i.e., a do little-to-nothing approach, that may assist them in regulating their response to external events, such as lockdowns, thereby facilitating a more positive outlook and experience [25,26]. Together, these findings suggest that older adults are more resilient and protected from the acute negative phycological impact of the COVID-19 lockdown. In contrast to similar studies [28,29], older individuals are well represented in our study, with 15% of our study population over 70 years old and a further 44% of our study population over 50 years old. The oldest individuals in our study were 90 years old.

We also observed an association between education level and happiness. Specifically, individuals who received a postgraduate education (i.e., graduated from a masters or PhD program) reported positive associations with happiness, but intriguingly, education level had no influence on anxiety. It is possible that postgraduate education is associated with job security and stability, as those individuals are more likely to be employed in knowledge-based sectors that can readily transition to working-from-home during the COVID-19 pandemic under stay-at-home orders. According to the Australian Bureau of Statistics, individuals with postgraduate degrees were most likely to be employed in a job relevant to their degrees [30] and receive a higher median salary [31] compared to their undergraduate or high-school counterparts. Alternatively, those with a postgraduate education may be more adept in critical analysis of information released by news media such that the perception of being more knowledgeable resulted in a greater perception of happiness [32]. Interestingly, Kantor et al. also noted that media consumption in the general US population was not associated with the presence of anxiety, irrespective of adjustments for education level [33]. Thus, the association between education and emotional resilience remains to be elucidated.

Another important variable that significantly influenced perceptions of happiness and anxiety in our study was worry about being lonely. We found that worry about loneliness was strongly associated with lower levels of happiness and higher levels of anxiety, and, more importantly, that it was independent of age and gender. In contrast, loneliness has been described by several studies as a by-product of social isolation during the COVID-19 pandemic that is strongly influenced by age and gender [4,34,35,36,37]. These studies consistently describe loneliness as being highest amongst young adults and lowest amongst older adults over 60 years old, and furthermore—in contrast to our findings—highest in females. Our results likely reflect the short-term impact of lockdown compared to the persistent social isolation and high rates of SARS-CoV-2 infections reported in those studies. In other studies, young adults reported feelings of increasing loneliness due to a reduction in the quantity of their social interactions during the COVID-19 pandemic. Older adults tend to value quality rather than quantity in their social networks, and young people tend to move more in their friendship networks and have a larger number of interactions [38].

Additionally, loneliness in the context of COVID-19 has been associated with depression, anxiety, stress, and suicidal ideation [4,33,39]. It should be noted here that our loneliness measure was associated with whether an individual worried about becoming lonely rather than a direct report of their current feeling of loneliness. It is conceivable from the data we presented that happiness, anxiety, and loneliness, as risk factors of psychological distress, may have interacting and bi-directional effects.

We also found that participants in regional and remote Australia who were worried about becoming lonely were reportedly less happy than participants in the major cities who also worried about becoming lonely during the pandemic. This is seemingly counterintuitive, as regional and remote locations may be perceived to be more protected from COVID-19 given the low population density and ability to social distance. However, this may well be a double-edged sword in that remote and regional access to healthcare and medical resources in the event of an outbreak is limited [40,41]. Moreover, Australian rural and regional communities carry higher rates of co-morbid health issues that further predispose individuals to COVID-19 [42]. It is also possible that the livelihood of many remote and rural Australians is dependent on transient economics (e.g., tourism and fly-in-fly-out workers in mining), which effectively ceased during the period of lockdown, contributing to reduced feelings of happiness despite anxiety levels remaining low.

Our research captured a large, representative sample of the adult Australian population across age, sex, education, and rurality. Limitations of this study include responses arising from a cross-sectional snapshot of the Australian population, where causation cannot be inferred. While we report on several significant findings, we cannot infer causation or direction of associations between happiness, anxiety, worry, age, education, and geographical location. However, the cross-sectional design of this study does lend support towards identifying potential interactions and suggests that younger individuals, individuals with lower levels of education, those worrying about loneliness, and those living outside of major cities are at increased risk of experiencing lower levels of happiness and increased stress responses. We also note that the smaller number of responses from populations living in regional, rural, and remote areas may bias the statistical outcomes of the measured interactions; however, the survey responses received are in proportion to and reflective of the Australian population density in the various regions. We also recognize that the measures of happiness, anxiety, and worry are self-reported and therefore subjective. However, self-cognitions are often assessed using single-item questions to reliably capture a global reference for individual self-assessed cognitions. There is a vast amount of literature using self-reports of psychosocial cognitions using a single item. For example, a single-item question of happiness was positively associated with older age and negatively correlated with anxiety, indicative of a valid tool for population studies [43,44].

## 5. Conclusions

We examined whether age, literacy, and geographic location influenced the feeling of happiness or anxiety. Furthermore, we assessed whether worrying about becoming lonely, as a factor of loneliness, was associated with self-cognitions of happiness or anxiety. This dataset provided a better understanding of factors that influence psychological well-being and quality of life in the Australian population and determined whether happiness may be an associative factor that could mitigate self-feelings of isolation and anxiety. Our study also provides insights into the evident negative impact of COVID-19 on rural youth and those less educated, with reduced happiness and increased anxiousness as factors. Worrying about becoming lonely in rural areas during the COVID-19 pandemic may adversely affect the well-being of rural populations. Preventions and supportive programs that focus on interventions to improve these negative social cognitions are worthy of consideration during subsequent COVID-19 lockdowns.

## Figures and Tables

**Table 1:** Sample characteristics.

Characteristic	N	%
**Population**	1593	100
**Age**		
18–29	348	21.8
30–39	299	18.8
40–49	244	15.3
50–59	245	15.4
60–69	223	14.0
70–79	183	11.5
80 and over	51	3.2
**Sex**		
Female	825	51.8
Male	764	48.0
Missing	4	0.3
**Education**		
High school	427	26.8
Undergraduate or equivalent degree	902	56.6
Postgraduate degree	228	14.3
Missing	36	2.3
**Rurality**		
MM1 (major city)	1275	80.0
MM2 (regional)	147	9.2
MM3-4 (rural)	71	4.5
MM5-7 (remote)	100	6.3

MM refers to Modified Monash Model of rurality: MM1 (urban/city), MM2 (regional), MM3-4 (rural), MM5-7 (remote).

**Table 2:** General linear regression modelling of factors associated with happiness.

	Reference	Variable	Model 1: Demographics		Model 2: Demographics + Loneliness		Model 3: Demographics + Loneliness + Rurality	
			Estimate	95% CI	Estimate	95% CI	Estimate	95% CI
Age	18–29	30–39	0.29	(−0.09–0.66)	0.34	(−0.02–0.71)	0.37	(0.01–0.73) *
		40–49	0.21	(−0.18–0.61)	0.07	(−0.32–0.45)	0.08	(−0.31–0.46)
		50–59	0.31	(−0.09–0.71)	0.08	(−0.31–0.46)	0.07	(−0.31–0.45)
		60–69	1.04	(0.63–1.46) **	0.77	(0.37–1.17) **	0.77	(0.37–1.17) **
		70–79	1.66	(1.22–2.1) **	1.29	(0.86–1.72) **	1.31	(0.88–1.74) **
		80 and over	1.73	(1.01–2.45) **	1.46	(0.76–2.16) **	1.41	(0.71–2.1) **
Sex	Female	Male	−0.03	(−0.28–0.21)	−0.03	(−0.27–0.2)	−0.03	(−0.27–0.2)
Education	High school degree	Undergraduate or equivalent	0.07	(−0.21–0.35)	0.03	(−0.25–0.3)	0.03	(−0.24–0.3)
		postgraduate	0.59	(0.19–0.99) **	0.69	(0.3–1.08) **	0.69	(0.3–1.08) **
Rurality	MM1 (major city)	MM2 (regional)	−0.25	(−0.66–0.17)	−0.34	(−0.74–0.07)	−0.01	(−0.47–0.45)
		MM3-4 (rural)	0.05	(−0.52–0.63)	−0.22	(−0.78–0.34)	−0.28	(−0.87–0.31)
		MM5-7 (remote)	−0.29	(−0.78–0.2)	−0.32	(−0.79–0.16)	0.07	(−0.48–0.63)
Loneliness	Loneliness (not worried)	Loneliness (worried)	-	-	−1.58	(−1.84–−1.32) **	−1.42	(−1.69–−1.14) **
Rurality and Loneliness Interactions	Interaction: MM1 and loneliness	Interaction: MM2 and loneliness	-	-	-	-	−1.38	(−2.34–−0.42) **
		Interaction: MM3-4 and loneliness	-	-	-	-	0.90	(−0.8–2.59)
		Interaction: MM5-7 and loneliness	-	-	-	-	−1.40	(−2.45–−0.34) **

Records with missing outcome or covariates were excluded. * *p* < 0.05; ** *p*< 0.01. MM refers to Modified Monash Model of rurality: MM1 (urban/city), MM2 (regional), MM3-4 (rural), MM5-7 (remote).

**Table 3:** General linear regression modelling of factors associated with anxiety.

	Reference	Variable	Model 1: Demographics		Model 2: Demographics + Loneliness		Model 3: Demographics + Loneliness + Rurality	
			Estimate	95% CI	Estimate	95% CI	Estimate	95% CI
Age	18–29	30–39	0.37	(−0.07–0.81)	0.27	(−0.15–0.68)	0.26	(−0.15–0.68)
		40–49	−0.30	(−0.76–0.16)	−0.16	(−0.6–0.28)	−0.16	(−0.6–0.27)
		50–59	−0.85	(−1.31–−0.38) **	−0.54	(−0.98–−0.1) *	−0.53	(−0.97–−0.09) **
		60–69	−1.37	(−1.85–−0.89) **	−1.02	(−1.48–−0.56) **	−1.02	(−1.48–−0.56) **
		70–79	−1.88	(−2.39–−1.36) **	−1.45	(−1.94–−0.95) **	−1.46	(−1.95–−0.97) **
		80 and over	−1.08	(−1.93–−0.24) **	−0.85	(−1.65–−0.05) **	−0.84	(−1.64–−0.03)*
Sex	Female	Male	−0.18	(−0.47–0.1)	−0.14	(−0.41–0.12)	−0.14	(−0.41–0.13)
Education	High school degree	Undergraduate or equivalent	−0.04	(−0.37–0.29)	0.06	(−0.25–0.37)	0.06	(−0.25–0.37)
		postgraduate	−0.18	(−0.65–0.29)	−0.27	(−0.72–0.18)	−0.27	(−0.71–0.18)
Rurality	MM1 (major city)	MM2 (regional)	−0.25	(−0.74–0.24)	−0.10	(−0.57–0.36)	−0.18	(−0.71–0.35)
		MM3-4 (rural)	−0.76	(−1.44–−0.08)*	−0.40	(−1.04–0.24)	−0.39	(−1.07–0.29)
		MM5-7 (remote)	−0.50	(−1.08–0.08)	−0.39	(−0.93–0.16)	−0.59	(−1.23–0.05)
Loneliness	Loneliness (not worried)	Loneliness (worried)	-	-	2.22	(1.93–2.51) **	2.16	(1.84–2.47) **
Rurality and Loneliness Interactions	Interaction: MM1 and loneliness	Interaction: MM2 and loneliness	-	-	-	-	0.31	(−0.79–1.41)
		Interaction: MM3-4 and loneliness	-	-	-	-	−0.22	(−2.17–1.73)
		Interaction: MM5-7 and loneliness	-	-	-	-	0.73	(−0.48–1.94)

Records with missing outcome or covariates were excluded. * *p* < 0.05; ** *p*< 0.01. MM refers to Modified Monash Model of rurality: MM1 (urban/city), MM2 (regional), MM3-4 (rural), MM5-7 (remote).

## Data Availability

The data presented in this study may be available upon reasonable request from the authors. The data are not made publicly available due to ethical considerations of participant details.
